# Thyrotoxicosis due to Gestational Trophoblastic Disease: Unmet Needs in the Management of Gestational Thyrotoxicosis

**DOI:** 10.1155/2024/5318871

**Published:** 2024-08-29

**Authors:** Kalyan Mansukhbhai Shekhda, Vladislav Zlatkin, Bernard Khoo, Eleni Armeni

**Affiliations:** ^1^ Department of Diabetes and Endocrinology Royal Free Hospital, London, UK; ^2^ University College London, London, UK; ^3^ School of Health Sciences University of Birmingham, Birmingham, UK

## Abstract

Thyrotoxicosis during pregnancy is rare but can have severe adverse consequences for the mother or foetus if left undiagnosed and untreated. It can be caused by an underlying thyroid disease or develop as gestational transient thyrotoxicosis. Molar pregnancy stands out as a pathological condition characterized by abnormal trophoblastic cell growth, which can manifest in benign or malignant forms, and is diagnosed with a disproportionate elevation of *β*-hCG (beta-human chorionic gonadotrophin) and specific features on ultrasonography including absent sac and large multicystic or honeycomb appearance. A pronounced increase in *β*-hCG levels can trigger hyperthyroidism, due to the structural resemblance between *β*-hCG and thyroid-stimulating hormone (TSH), although the thyrotrophic effects of *β*-hCG could vary between patients diagnosed with gestational trophoblastic disease (GTD). In this report, we present two cases (Patient 1: 43 years, Patient 2: 31 years) who came to emergency department following a history of vaginal spotting, palpitations, and hyperemesis. In both patients, blood tests indicated disproportionately elevated *β*-hCG levels along with high levels of Free T4 (FT4) and Free T3 (FT3), as well as suppressed TSH levels. Ultrasonography showed nonviable products of conception with large multicystic hemorrhagic lesions and empty gestational sacs, thereby confirming GTD. The Burch–Wartofsky Point Scale scores were 20 and 15 points, respectively, suggesting that they were less likely to be in thyroid storm at presentation. Antithyroid medications were administered, followed by evacuation of the products of conception. Postoperatively, their thyroid function was normalized. These cases underscore the importance of ruling out thyroid storm, monitoring thyroid function, and treating hyperthyroidism appropriately before undergoing surgical treatment. It is also important to consider the variability in the thyrotrophic effects of *β*-hCG among individuals diagnosed with GTD. In addition to monitoring free thyroid hormone levels, it is crucial to consider clinical symptoms to effectively manage such cases.

## 1. Introduction

Although rare, identifying and treating hyperthyroidism during pregnancy is essential to prevent adverse outcomes for the mother or the foetus [1]. Gestational transient thyrotoxicosis (GTT) is characterized by nonautoimmune transient hyperthyroidism in pregnant women due to excessive production of thyroid hormones secondary to the thyrotrophic effects of *β*-hCG (beta-human chorionic gonadotrophin) [2]. During pregnancy, GTT is more common compared to Graves' disease with a prevalence of GTT around 2%–11% and Graves' disease around 0.1%–1% in all pregnancies. GTT usually lasts for a short period of time and resolves spontaneously after the first trimester or after treating the underlying cause [2].

Gestational trophoblastic disease (GTD) is one of the rare causes of GTT where excessive growth of extraembryonic trophoblastic cells due to abnormal placental development leads to the overproduction of thyrotrophic *β*-hCG. Due to the structural similarity of *β*-HCG to thyroid stimulating hormone (TSH), this molecule is capable of binding to the TSH receptor, allowing it to mimic high levels of TSH that cause biochemical hyperthyroidism [3]. GTD encompasses a spectrum of conditions ranging from benign to malignant, including hydatidiform mole (partial or complete), placental site trophoblastic tumors, and choriocarcinoma. The overall prevalence of GTD is approximately 1.2 per 1,000 pregnancies, with choriocarcinoma accounting for 0.5 per 1,000 cases [4]. Patients are usually present with large for dates uterus, vaginal bleeding, hyperemesis, and the diagnosis is confirmed by disproportionately elevated *β*-hCG for gestational age, typical honeycomb appearance on ultrasonography, absent foetal parts, and cystic appearance of placenta [5]. Due to rarity of GTD and different protocols for the management followed in different continents, association between GTT and GTD is highly variable worldwide [6]. In parts of western world (the US and the UK), prevalence of GTT is 4%–16% among patients with GTD, whereas it is higher in African and Iranian populations standing at 25%–50% of the patients with GTD [6]. The possible explanation for this trend could be early detection of GTT in patients with GTD, more robust pathways to treat GTT and GTD earlier and effectively, and more healthcare resources available in western countries [6]. Patients with complete mole (CM) are more likely to have a higher rate of biochemical GTT compared to partial mole (PM) [6]. The definitive treatment for GTD is surgical evacuation and curettage leading to spontaneous resolution of hyperthyroidism postoperatively. Up to 5% of patients with PM and up to 20% of patients with CM develop gestational trophoblastic neoplasia, which is the severe form of GTD, and require specialist review and treatment such as chemotherapy or radiotherapy [5].

We present two cases of GTT due to GTD with variable symptoms and hyperthyroidism.

## 2. Case Series

### 2.1. Case 1

A 43-year-old pregnant woman came to emergency department with vaginal spotting and palpitations for 5 days. She had a history of amenorrhea for 10 weeks prior to the presentation and her *β*-hCG levels were disproportionately elevated for the estimated gestational age. Ultrasound revealed a large multicystic/hemorrhagic lesion within the endometrium, measuring 82 mm × 110 mm × 66 mm, without evidence of gestation sac: findings suggestive of molar pregnancy. Her biochemical profile is presented in Table 1. Her Burch–Wartofsky Point Scale (BWPS) score was 20, suggesting that the patient was less likely to be in thyroid storm (BWPS score of >45 suggestive of thyroid storm, BWPS score between 25 and 45 highly suggestive of impending thyroid storm, BWPS score < 25 suggest less likely to be thyroid storm). Her thyroid receptor antibodies were negative which ruled out Graves' disease.

**Figure 1:**
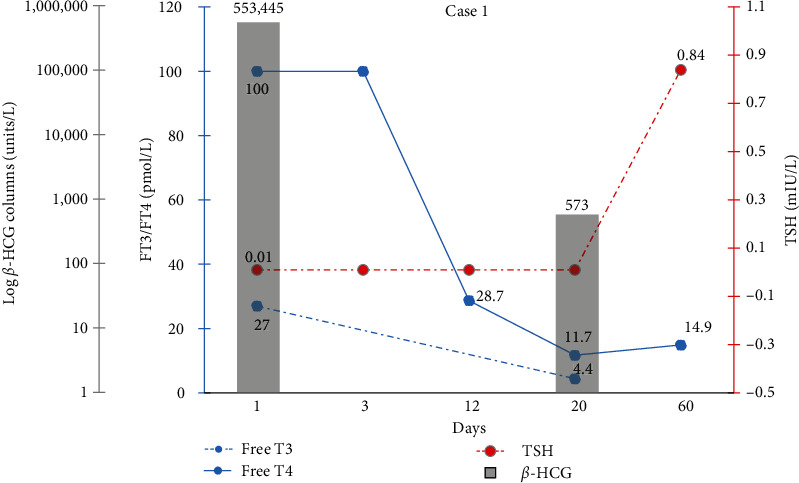
First patient's *β*-HCG and thyroid function tests in the first patient over a 60-day period. Final follow-up occurred on day 60. Postevacuation *β*-HCG was taken on day 20.

The patient was given carbimazole 60 mg OD orally for 2 days and propranolol 40 mg before surgical evacuation of molar pregnancy. Carbimazole was stopped postoperatively. Histology confirmed complete hydatidiform molar pregnancy, resolution of which was later confirmed on down-trending follow-up *β*-hCG and thyroid function test. The patient was referred to the specialist oncology unit for further assessment and consideration of further treatment such as chemotherapy if required. Her latest available *β*-hCG levels were 573 IU/L with a normal thyroid function test. The change in hormone levels from the time of presentation to the time of resolution is presented in Figure 1.

### 2.2. Case 2

A 31-year-old female presented to the emergency department with vomiting, headache, dizziness, and lethargy for 1 week. She had a history of amenorrhea for 12 weeks prior to admission and her *β*-hCG levels were disproportionately elevated for the estimated gestational age. Her biochemical profile is depicted in Table 1. Ultrasonography showed an absent gestational sac with a distended hyperechoic endometrial tissue without vascularity. Her BWPS score was 15 and her thyroid receptor antibodies were negative which ruled out Graves' disease.

**Figure 2:**
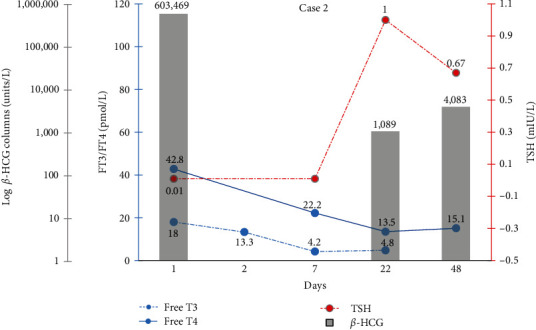
Second patient's *β*-HCG and thyroid function tests trend over a period of 48 days. Follow-up occurred on day 48, when a choriocarcinoma was discovered. Postevacuation *β*-HCG was taken on day 22.

The patient was started on propylthiouracil 150 mg twice a day with propranolol 20 mg twice a day for 2 days. She underwent evacuation of the product of conception, postoperative period was uneventful, and medications were stopped. Histology confirmed the diagnosis of partial hydatiform molar pregnancy. During follow-ups, her *β*-HCG levels remained slightly elevated, at 1,089 IU/L, which later was investigated, and she underwent repeat evacuation where progression to choriocarcinoma was found on histology. At this point, her *β*-HCG levels were 4,083 IU/L. She was then referred to a specialist center for further management and consideration for chemotherapy, where she was started on long-term daptomycin. Changes in hormone levels from the time of presentation until the time of diagnosis of choriocarcinoma are presented in Figure 2.

The laboratory assessment of Case 2 showed a higher level of *β*-hCG compared to Case 1, but the thyrotoxicosis was not as severe.

Table 1 compares the demographic, clinical, and biochemical differences between these patients.

## 3. Discussion

GTT is a prevalent phenomenon seen in pregnancy, primarily due to the structural similarity between *β*-HCG and TSH beta subunits, as well as their shared alpha subunit [7]. This structural resemblance enables hCG to bind to the TSH receptor, thus mimicking the effects of TSH [7]. GTT generally occurs in the first trimester of pregnancy and resolves by 14–18 weeks of gestation when *β* -HCG values decrease [8]. It is important to differentiate it from Graves' disease (GD), another cause of hyperthyroidism in pregnancy, because of different management. Due to normal changes in physiology during pregnancy leading to heat intolerance, anxiety, emotional lability, nausea, and vomiting, it may be difficult to differentiate between normal changes and GTT or Graves' disease. More attention should be directed to look for more specific signs such as tremors, weight loss despite normal or increased appetite, exophthalmos, or signs of heart failure that point toward diagnosis of GD in pregnancy [8]. TSH receptor antibodies are specific for GD and should be tested [8]. Both of our patients neither have specific clinical signs nor TSH receptor antibodies were positive, ruling out this cause. GTT can rarely be caused by GTD which can be diagnosed based on history (vaginal bleeding in early pregnancy, history of GTD in the past) and investigation results indicating a disproportionately raised *β*-HCG and features of GDT on ultrasonography [5]. Additionally, GTT in patients with GTD is not transient if surgical management is not timely. People with hyperthyroidism in pregnancy can rarely present with severe thyrotoxicosis; therefore, it is important to look for specific signs, such as atrial fibrillation, heart failure, and liver dysfunction.

It is hypothesized that thyrotropic activity of *β*-HCG in women with GTD is higher compared to those with normal pregnancies. This claim is corroborated by the observation that *β*-HCG associated with GTD induces a more substantial increase in cAMP production when introduced to the human thyroid stimulating hormone receptor (TSH-R) in Chinese hamster ovary cells [9]. Moreover, the thyrotropic activity of *β*-HCG might be influenced by the metabolism of the *β*-HCG molecule; alterations such as deglycosylation or desialylation could potentially enhance its ability to stimulate the thyroid gland [3].

Both patients in our study presented with hyperthyroidism and GTD, exhibiting varying clinical features. The patients received different treatments due to the varied clinical signs of thyrotoxicosis, different biochemical results, and the lack of a standardized protocol to manage hyperthyroidism in GTD. Both patients received antithyroid medications for 2 days before surgical evacuation. Though none of them developed any complications related to hyperthyroidism postoperatively, these cases underscore the complex clinical dilemmas faced in the care of patients with biochemical hyperthyroidism but lack clear clinical indications of thyrotoxicosis in early pregnancy. The review of similar cases published in the literature is described in Table 2, which shows that nonstandardized treatment protocols have been followed to treat hyperthyroidism.

Patients with GTD should be managed under a multidisciplinary team including an obstetrician, endocrinologist, intensive care physician, and anaesthesiologist. Although they are usually treated with the surgical evacuation of gestational products, it is very important to rule out and treat thyroid storms and optimize their thyroid function before surgical intervention as untreated hyperthyroidism or thyroid storm can trigger further deterioration in their condition and has significantly high morbidity and mortality [15].

A Japanese survey found a mortality rate over 10% in thyroid storm patients, with multiorgan and acute heart failure being the leading causes of death [15]. Diagnosing thyroid storm requires clinical suspicion and validated scores like the BWPS > 45 or Japanese Thyroid Association (JTA) categories TS1 or TS2 [15, 16]. BWPS and JTA criteria have shown overall agreement, but BWPS > 45 may select more patients for aggressive therapy. Both systems should be used together to enhance diagnostic accuracy [17].

Medical treatment includes antithyroid medications, steroids, beta-blockers, and inorganic iodide. The American Thyroid Association (ATA) recommends propylthiouracil (PTU) over methimazole (MMI) for rapidly decreasing triiodothyronine (T3) levels, with a PTU dose of 600 mg/day or MMI 60 mg/day, whereas JTA recommends using either of this as national wide survey conducted in Japan did not find any significant difference in severity and mortality outcomes in patients treated with MMI or PTU [15]. Both guidelines suggest that corticosteroids, individualized in dose, should be used to address adrenal insufficiency and thyrotoxicosis, but our patients did not receive them. Generally, hydrocortisone 100 mg every 8 hr or dexamethasone 8 mg/day IV is recommended [17]. For beta-blockers, ATA suggests propranolol 60–80 mg every 4 hr, while JTA prefers selective *β*-1 blockers like esmolol or bisoprolol. As per the recent prospective study, rapid administration of Inorganic iodide should be considered in iodine-sufficient patients with thyroid storm, as it helps with rapid reduction in thyroid hormone levels and reduction in fatality rate in these patients [17, 18].

After treatment with surgical intervention, patients should undergo frequent *β*-hCG level monitoring until suppressed below 5 IU/L, under the guidance of the gynecological team, and then followed up to rule out resurgence. From an endocrinology perspective, the thyroid function test should be monitored until autoimmune hyperthyroidism is ruled out and a downward trend of *β*-HCG has been confirmed together with the resolution of biochemical hyperthyroidism has been confirmed [10].

## 4. Conclusion

In conclusion, as seen in our cases, due to a lack of standardized protocol for the management of GTT, different treatment protocols were followed. In the end, neither patient had lasting endocrine complications, and postoperative recovery was uneventful. A combined evaluation by a multidisciplinary team including endocrinology and obstetrics is required for the management of patients with GTT secondary to GTD. Given the different effects of hCG on thyroid hormone levels, the decision about treatment should be guided primarily by the clinical signs and the state of the patient. A thorough assessment of vital signs and symptomatology will minimize the risk of thyroid emergencies during the upcoming surgical intervention.

## Figures and Tables

**Table 1:** Comparison of demographic data, clinical characteristics, and blood results on admission.

Characteristic	Case 1	Case 2
Age	Forty-three years	Thirty-one years
Previous pregnancies	Four normal vaginal deliveries Two caesarean sections	One caesarean section
Last pregnancy	Two years ago	Two years ago
Previous history of gestational trophoblastic disease or molar pregnancy	No	No
Previous miscarriages	Two miscarriages (10 and 15 years ago)	None
Past medical history	Hypertension	No past medical history
Medications	Labetalol	Not on any medications
Symptoms	Vaginal spotting	Vomiting, dizziness
Burch–Wartofsky score	20	15
Pulse rate	98/min	90/min
Blood pressure	149/96 mmHg	118/70 mmHg
*β*-hCG	553,445 units/L	603,439 units/L
TSH (RR: 0.3–4.2 munit/L)	<0.01 mIU/L	<0.01 mIU/L
Free T4 (RR: 12–22 pmol/L)	>100 pmol/L	42.8 pmol/L
Free T3 (RR:3.1−6.8 pmol/L)	28.6 pmol/L	18.0 pmol/L
TSH receptor antibodies (RR: 0–0.4 units/L)	<0.4 units/L	<0.4 units/L

*β* -hCG reference ranges for gestational age as below 3 weeks: 5–72 units/L, 4 weeks: 10−708 units/L, 5 weeks: 217–8,245 units/L, 6 weeks: 152–32,177 units/L, 7 weeks: 4,059–153,767 units/L, 8 weeks: 31,366–149,094 units/L, 9 weeks: 59,109–135,901 units/L, 10 weeks: 44,186–170,409 units/L, and 12 weeks: 27,107–201,165 units/L.

**Table 2:** Case reports of hyperthyroidism due to gestational trophoblastic diseases (GTD).

Author, Year	Study design	Presenting symptoms	BWPS	Biochemistry				Management		Outcome and follow-up
				*β*-HCG (units/L)	TSH (mIU/L)	FT4 (pmol/L)	FT3 (pmol/L)	Hyperthyroidism	GTD	
Blick and Schreyeral, 2019 [10]	Case report	Vaginal bleeding, abdominal pain	55	117,495	<0.01	27.95	NA	PTU 500 mg Methylprednisolone 80 mg Propranolol 2 mg	D&C	Euthyroidism, uneventful

Hodgson et al., 2021 [11]	Case report	Vaginal bleeding, fatigue, and abdominal distention	NA	219,687	NA	NA	NA	No treatment given for hyperthyroidism preoperatively	D&C	Postoperative thyroid storm due to undiagnosed hyperthyroidism preoperatively

Da Silva Santo et al., 2022 [12]	Case report	Vaginal bleeding, nausea, and vomiting	NA	978,485	<0.01	77.86	10.41	PTU 150 mg TDS Propranolol 40 mg TDS Dexamethasone 8 mg OD	D&C followed by methotrexate	Euthyroidism, Invasive mole

Grzechocinska et al., 2021 [13]	Case report	Vaginal bleeding	NA	414,937	<0.01	60.7	16	Thiamazole 20 mg TDS Hydrocortisone 100 mg QDS	D&C	Euthyroidism, uneventful

Saleem et al., 2021 [14]	Case report	Fever, headache, and vomiting	65	3,058,000	<0.01	61	27.49	Propranolol 20 mg TDS Dexamethasone 10 mg TDS Carbimazole 15 mg BD Cholestyramine 4 mg QDs Lugol's iodine	Chemotherapy (methotrexate)	Euthyroidism, metastatic choriocarcinoma

Our cases	Case 1	Vaginal spotting, palpitations	15	553,445	<0.01	>100	28.6	Carbimazole 60 mg OD Propranolol 40 mg BD	D&C	Euthyroidism, uneventful
	Case 2	Vomiting, headache, and dizziness	15	603,439	<0.01	42.8	18.0	PTU 150 mg BD, propranolol 20 mg BD	D&C	Euthyroidism, choriocarcinoma

NA, not available; *β*-HCG, *β*-human chorionic gonadotrophins; TSH, thyroid stimulating hormone; FT4, free T4; FT3, free T3; GTD, gestational trophoblastic disease; PTU, propylthiouracil; D&C, dilatation & curettage; and BWPS, Burch–Wartofsky Point Scale.

## Data Availability

Data used to support the findings of this study are included in the article.
